# Studies on Antifungal Properties of Methacrylamido Propyl Trimethyl Ammonium Chloride Polycations and Their Toxicity *In Vitro*

**DOI:** 10.1128/spectrum.00844-23

**Published:** 2023-05-11

**Authors:** Magdalena Skóra, Magdalena Obłoza, Małgorzata Tymecka, Bartlomiej Kalaska, Magdalena Gurgul, Kamil Kamiński

**Affiliations:** a Department of Infections Control and Mycology, Chair of Microbiology, Jagiellonian University Medical College, Cracow, Poland; b Department of Pharmacodynamics, Medical University of Białystok, Białystok, Poland; c Faculty of Chemistry, Jagiellonian University, Cracow, Poland; d Doctoral School of Exact and Natural Sciences, Faculty of Chemistry, Jagiellonian University, Cracow, Poland; University of Iowa Hospitals and Clinics

**Keywords:** polycations, antifungal, multidrug resistant, molecular mass

## Abstract

The biological activity of polycations is usually associated with their biocidal properties. Their antibacterial features are well known, but in this work, observations on the antifungal properties of macromolecules obtained by methacrylamido propyl trimethyl ammonium chloride (MAPTAC) polymerization are presented. The results, not previously reported, make it possible to correlate antifungal properties directly with the structure of the macromolecule, in particular the molecular mass. The polymers described here have antifungal activity against some filamentous fungi. The strongest effect occurs for polymers with a mass of about 0.5 mDa which have confirmed activity against the multidrug-resistant species Scopulariopsis brevicaulis, Fusarium oxysporum, and Fusarium solani, as well as the dermatophytes Trichophyton mentagrophytes, Trichophyton rubrum, Trichophyton interdigitale, and Trichophyton tonsurans. In addition, this publication describes the effects of these macromolecular systems on serum and blood components and provides a preliminary assessment of toxicity on cell lines of skin-forming cells, i.e., fibroblasts and keratinocytes. Additionally, using a Franz diffusion chamber, a negligibly low transport of the active polymer through the skin was demonstrated, which is a desirable effect for externally applied antifungal drugs.

**IMPORTANCE** Infectious diseases are a very big medical, social, and economic problem. Even before the COVID-19 pandemic, certain infections were among of the most common causes of death. The difficulties in the treatment of infectious diseases concern in particular fungal diseases, against which we have only a few classes of drugs represented by a few substances. The publication presents the preliminary results of the *in vitro* antifungal activity studies of four MAPTAC polymers on different fungal species and their cytotoxicity to human cells (fibroblasts and keratinocytes). The paper also compares these properties with analogous ones of two commonly used antifungal drugs, ciclopirox and terbinafine.

## INTRODUCTION

Infectious diseases are a very big medical, social, and economic problem. Even before the COVID-19 pandemic, certain infections were among of the most common causes of death. According to World Health Organization (WHO), in 2019 lower respiratory infections were the fourth leading cause of death globally (https://www.who.int/news-room/fact-sheets/detail/the-top-10-causes-of-death). Many infectious diseases are highly contagious and spread easily in the population. Access to effective methods of preventing and treating these diseases is therefore a very important element of taking care of public health. The severe acute respiratory syndrome coronavirus 2 (SARS-CoV-2) pandemic has shown how serious the consequences of the lack of appropriate prevention and treatment of infectious diseases can be (1).

Treatment of infections presents many difficulties. The greatest challenges are the relatively small number of possible drugs and the few families of biologically active chemical structures practically available. Additionally, the chances of a successful cure are reduced by resistance of the pathogens to these drugs and their side effects and contraindications for use. Drug resistance is one of the biggest problems that pharmaceutical and related sciences are facing in the 21st century so far. There have been no developed effective treatments for all major infectious diseases, and the prospect of drug resistance means that the available tools may soon become ineffective (2). The mechanisms of drug resistance are not fully known, and some have postulated that the reason may not be as straightforward as simply the overuse of antimicrobials (2, 3). Drug resistance processes are also caused by factors outside the human body related to environmental pollution, such as heavy metals in the soil (3, 4).

The difficulties in the treatment of infectious diseases concern in particular fungal diseases (5), against which there are only a few classes of drugs represented by a few substances (6). Mycoses in the general population occur quite frequently (e.g., onychomycosis and candidiasis of the mucous membranes) but the course of those diseases is usually not dramatic. Life-threatening infections affect specific groups of patients with certain predisposing factors, i.e., profound immunodeficiencies. Due to the great biodiversity of fungi, some infections occur infrequently and are even considered rare diseases (https://www.orpha.net/consor/cgi-bin/index.php?lng=EN). These circumstances mean that fungal infections are not of interest to the global health policy and are treated neglectfully by “Big Pharma.” However, mycoses could be serious complications of some very common diseases, i.e., diabetes, or their therapy, e.g., immunosuppressive and cytostatic treatment and steroid and antibiotic therapies. Furthermore, fungal-viral coinfections also prove to be dangerous, especially as the COVID-19 outbreak demonstrated (7, 8). In this light, the rank of fungal infections changes. There is a great need to improve the effectiveness of combating fungal diseases. The best solution seems to be the creation of new structures that will use new mechanisms of molecular action not yet known in drug design (9). In this investigation, polymers with a positive charge were used for this purpose. Polymers are substances which have made probably the strongest contribution to humans’ everyday lives over the last hundred years (10). The technologies enabling their synthesis and processing have given widespread access to inexpensive clothing, food packaging, and materials widely used in medicine. The specific properties of macromolecules widely present in living organisms and critical to their function (proteins, nucleic acids, and polysaccharides) suggest the relevance of studying synthetic bioactive macromolecules to obtain polymeric drugs (11). Biocidal properties of positively charged macromolecules are widely known, especially antibacterial action (12). This effect is thought to result mainly from electrostatic interactions between the negatively charged biological membrane and the positively charged polycations (13, 14). Similar interactions are also often observed with proteins and other biopolymers present in the living organism, which are predominantly negatively charged. The product of these interactions are often aggregates of submicrometric-sized particles with biological activity different from that of the free biomacromolecules involved in these processes. An example of such a purposeful process is the neutralization of the anticoagulant heparin (15), but these reactions are in most cases undesirable and occur after administration of polycations into the bloodstream. These phenomena are responsible for most of the negative effects of polycation use (16), but they could theoretically also generate other potentially desirable biological properties, so it makes sense to examine the selectivity of these interactions.

**FIG 1:**
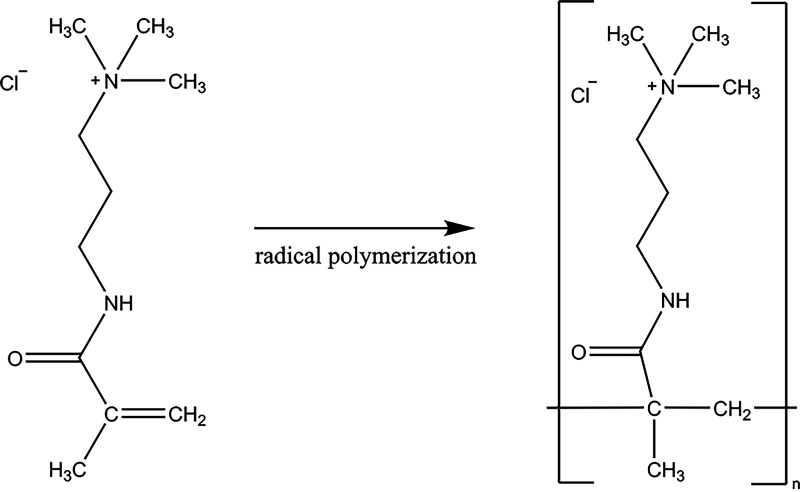
Polymerization of methacrylamido propyl trimethyl ammonium chloride (MAPTAC) monomer.

This work aimed to compare the toxicity of synthetic macromolecules based on the methacrylamido propyl trimethyl ammonium chloride (MAPTAC) monomer (a simple methacrylate structure obtained by radical polymerization with a stable quaternary amine) (Fig. 1) of different masses against healthy mammalian and fungal cells to assess the possibility of using these structures against fungal diseases.

This polymer is widely reported in the scientific biomedical literature as a cationic component of block polymers (17) and hydrogels (18), as well as homopolymers on their own. Application for the synthesis of structures with a precisely defined molecular weight is possible thanks to the property of the MAPTAC monomer which allows it to be polymerized not only by classical radical polymerization but also by controlled radical reversible addition—fragmentation chain-transfer (RAFT) polymerization. Due to these properties, it has been used in this work which, by design, requires systems with defined molecular weights. There is a lack of systematic studies in the literature to assess the antifungal properties of positively charged macromolecules with a parallel toxicity assessment that could help to identify ways/places of their application in therapeutic practice. This work can change that. The publication presents the preliminary results of *in vitro* antifungal activity studies of four MAPTAC polymers with different fungal species and their cytotoxicity to human cells (fibroblasts and keratinocytes). The paper also compares these properties with analogous ones of two commonly used antifungal drugs, ciclopirox and terbinafine.

## RESULTS

### General physical and chemical characteristics of polymers.

The applied techniques and synthesis strategies made it possible to obtain macromolecules with a satisfactory range of molecular masses (exact values in Table 1; see also Fig. S3 in the supplemental material); thus, it will be possible to correlate this parameter with biological properties, which was the aim of this publication. The uncontrolled polymerization produced macromolecules with higher-molecular-weight dispersion (DP) values for single RAFT polymerization, as expected. However, the difference was not so significant that the polymers could not be compared. In addition, it was confirmed from the zeta potential values (exact values in Table 1) that the obtained macromolecules were polycations. The zeta potential values for polymers increased with increasing molecular weights (Table 1). The results of elemental analysis, ^1^H nuclear magnetic resonance (NMR), and Fourier transform infrared spectroscopy (FTIR) complement the results of molecular weight measurements and confirm the achievement of the intended polymer structures. Full data and their interpretation can be found in the supplemental material (Table S1 and Fig. S1 to S4).

**TABLE 1:** Physiochemical characteristics of the obtained polymers

Polymer^*a*^	Molecular weight, M	Dispersity index, DP	Zeta potential in water (mV)
PMAPTAC21	4.84	1.05	31.83 ± 5.35
PMAPTAC36	5.31	1.06	41.20 ± 5.17
PMAPTAC2.5k	549	1.25	61.9 ± 0.98
PMAPTAC4k	892	1.16	65.7 ± 3.72

a The number reflects the number of units calculated from the molecular weight obtained from GPC measurements [DP = (*M*_n_ − M_CTA_)/M_MAPTAC_ for RAFT polymerizations or DP = *M*_n_/M_MAPTAC_ for free radical polymerizations].

### Cell toxicity of polymers.

**(i) *In vitro* antimycotic properties.** The results of *in vitro* antifungal activity testing of PMAPTAC polymers indicate that molecules with higher molecular weights (about 0.5 mDa) exhibit greater antifungal properties than substances with lower weights. This antimycotic activity is selective and applies mainly to filamentous fungi (Tables 2 and 3). The studies show that the antifungal spectra of higher-molecular-weight polymers PMAPTAC4k and PMAPTAC2.5k are identical but the polymers differ in MIC values, which are lower, even several times, in the case of PMAPTAC4k (Table 3 and Tables S2 and S3). The obtained MIC values allowed us to select PMAPTAC4k as the compound with the best antifungal properties among the tested polymers and to subject it to wider research.

**TABLE 2:** Results of the preliminary studies on the antifungal activities of four polymers formed by MAPTAC polymerization showing different molecular masses

Fungal strain	MIC (μg/mL)			
	PMAPTAC21	PMAPTAC36	PMAPTAC2.5k	PMAPTAC4k
Candida albicans ATCC 90028	>250	>250	>250	>250
Candida krusei ATCC 6259	>250	>250	>250	>250
Aspergillus flavus ATCC 204304	>250	>250	>250	>250
Aspergillus brasiliensis ATCC 16404	>250	>250	>250 (31.25^*a*^)	>250 (3.91^*a*^)
Trichophyton mentagrophytes ATCC 18484	>250	>250	31.25	1.95 (0.98^*a*^)
Fusarium solani clinical isolate 155	>250	>250	15.62	1.95
*Scopulariopsis brevicaulis* clinical isolate 34	7.81	3.91	31.25	3.91

a Weakening of fungal growth in comparison to growth control; no complete inhibition of growth.

**TABLE 3:** MICs of PMAPTAC2.5k and PMAPTAC4k against broad-spectrum fungal species determined in the visual (molds and yeasts) and automatic (yeasts) reading controls

Fungal species	MIC (μg/mL)			
	PMAPTAC4k		PMAPTAC2.5k	
	Visual reading	Automatic reading^*a*^	Visual reading	Automatic reading^*a*^
Yeasts				
Candida albicans ATCC 90028	>250	>250	>250	>250
Candida auris DSM 21092	>250	>250	>250	>250
Candida glabrata ATCC 15454	>250	>250	>250	>250
Candida krusei ATCC 6258	>250	>250	>250	>250
Candida parapsilosis DSM 5784	3.91 to 7.81	3.91	15.62	15.62
Candida tropicalis ATCC 1369	>250 (7.81^*b*^)	31.25	>250 (15.62^*b*^)	31.25
Cryptococcus neoformans ATCC 204092	3.91 to 7.81	1.95	31.25	ND^*c*^
Cryptococcus neoformans DSM 6973	3.91 to 7.81	1.95	15.62	15.62
Cryptococcus neoformans clinical isolate EN	1.95	0.98	31.25	0.98
Molds				
Aspergillus brasiliensis ATCC 16404	>250 (0.98^*b*^)	ND	>250 (31.25^*b*^)	ND
Aspergillus flavus ATCC 204304	>250	ND	>250	ND
Aspergillus fumigatus DSM 819	>250	ND	>250	ND
Aspergillus terreus DSM 1958	>250	ND	>250	ND
Fusarium graminearum DSM 1095	3.91	ND	15.62	ND
Fusarium oxysporum DSM 841	1.95 to 7.81	ND	31.25	ND
Fusarium proliferatum DSM 840	1.95	ND	62.5	ND
Fusarium solani DSM 1165	0.98 to 3.91	ND	15.62	ND
Fusarium solani clinical isolate P155	0.98 to 3.91	ND	15.62	ND
Fusarium verticillioides DSM 62264	3.91	ND	31.25	ND
Mucor indicus DSM 2185	>250 (15.62^*b*^)	ND	>250 (125^*b*^)	ND
Mucor mucedo DSM 809	31.25 to >250	ND	>250	ND
Rhizopus oryzae DSM 854	>250 (15.62^*b*^)	ND	>250 (250^*b*^)	ND
*Scopulariopsis brevicaulis* DSM 9122	1.95	ND	15.62	ND
*Scopulariopsis brevicaulis* clinical isolate S34	3.91	ND	31.25	ND
Dermatophytes				
Trichophyton interdigitale clinical isolate D251	0.98	ND	31.25	ND
Trichophyton interdigitale DSM 4176	0.98	ND	62.5	ND
Trichophyton mentagrophytes ATCC 18748	0.98	ND	31.25	ND
Trichophyton rubrum DSM 16111	1.95	ND	62.5	ND
Trichophyton tonsurans clinical isolate D258	0.98	ND	31.25	ND
Trichophyton tonsurans DSM 12285	1.95	ND	15.62	ND

a Automatic reading was performed with a Tecan microdilution plate reader.

b Weakening of fungal growth in comparison to growth, no complete inhibition of growth.

c ND, not determined.

The activity of PMAPTAC4k against yeast was demonstrated for Cryptococcus neoformans, Candida parapsilosis, and Candida tropicalis at concentrations of 0.98 to 7.81 μg/mL, 3.91 to 7.81 μg/mL, and 7.81 to >250 μg/mL, respectively, which resulted in complete or partial inhibition of fungal growth (Table 3 and Table S2). Similar concentrations have an antifungal effect on selected filamentous fungi: the molds Scopulariopsis brevicaulis (MIC range, 1.95 to 3.91 μg/mL) and five Fusarium *s*pecies (MIC range, 0.98 to 7.81 μg/mL), as well as dermatophytes belonging to the genus *Trichophyton* (MIC range, 0.98 to 3.91 μg/mL) (Table 3). The antimycotic properties of the PMAPTAC4k polymer against *Scopulariopsis* and Fusarium species turned out to be even higher than those of two antifungal drugs used in the treatment of mycoses, terbinafine and ciclopirox, for which MIC values ranged above 100 μg/mL and 12.5 to 25 μg/mL for Fusarium and 25 to 100 μg/mL and 12.5 μg/mL for *Scopulariopsis* (Table 4). PMAPTAC4k had stronger antifungal activity than that of ciclopirox against the dermatophyte *Trichophyton* (MIC value, 12.5 μg/mL) but weaker than that of terbinafine (MIC range, 0.19 to 0.78 μg/mL) (Table 4). The antifungal effect of PMAPTAC4k was also observed in relation to other molds, but this activity was definitely weaker. The polymer at concentrations equal to or greater than 15.62 μg/mL and 0.98 μg/mL reduced the growth of two tested representatives of *Mucorales* and Aspergillus brasiliensis, respectively. This molecule did not exhibit any activity against the other tested Aspergillus species: Aspergillus flavus, Aspergillus fumigatus, and Aspergillus terreus (Table 3).

**TABLE 4:** Antimycotic activities of PMAPTAC4k, ciclopirox, and terbinafine against different fungal species

Fungal strain	MIC (μg/mL)		
	PMAPTAC4k	Ciclopirox	Terbinafine
Aspergillus brasiliensis ATCC 16404	>250 (3.91^*a*^)	12.5	25
Aspergillus flavus ATCC 204304	>250	12.5	12.5
Aspergillus fumigatus DSM 819	>250	12.5	50
Aspergillus fumigatus clinical isolate SU56	>250	12.5	50
Aspergillus terreus DSM 1958	>250	12.5	12.5
Candida albicans ATCC 90028	>250	12.5	>100
Candida glabrata ATCC 15545	>250	12.5	>100
Candida krusei ATCC 6258	>250	12.5	>100
Cryptococcus neoformans clinical isolate EN	1.95	12.5	>100
Fusarium oxysporum clinical isolate 109	1.95	25 (12.5^*a*^)	>100
Fusarium oxysporum DSM 841	1.95	12.5	>100
Fusarium solani clinical isolate 155	1.95	25	>100
Fusarium solani DSM 1164	1.95	25 (12.5^*a*^)	>100
*Scopulariopsis brevicaulis* clinical isolate 34	1.95	12.5	100 (50^*a*^)
*Scopulariopsis brevicaulis* DSM 9122	1.95	12.5	25 (12.5^*a*^)
Trichophyton interdigitale clinical isolate 251	1.95	12.5	0.78
Trichophyton interdigitale clinical isolate D182	1.95	12.5	0.39
Trichophyton interdigitale DSM 4167	1.95	12.5	0.19
Trichophyton mentagrophytes ATCC 18748	1.95	12.5	0.78
Trichophyton rubrum DSM 16111	3.91	12.5	0.39
Trichophyton tonsurans DSM 12285	1.95	12.5	0.39

a Weakening of fungal growth in comparison to growth control; no complete inhibition of growth.

The presence of serum was found to reduce the antifungal activity of the polymer PMAPTAC4k. Higher MIC values were obtained in *in vitro* studies with the strains grown in serum-supplemented medium than in serum-free medium (Table 5).

**TABLE 5:** Effect of serum on the antifungal activity of the polymer PMAPTAC4k *in vitro*

Fungal strain	MIC (μg/mL) in:			
	Medium without bovine serum	Medium with 80% bovine serum	Medium with 50% bovine serum	Medium with 20% bovine serum
Aspergillus brasiliensis ATCC 16404	>250 (1.95^*a*^)	>250	>250	>250
Aspergillus flavus ATCC 204304	>250	>250	>250	>250
Trichophyton mentagrophytes ATCC 18748	1.95	>250	>250	>250 (125^*a*^)
Trichophyton interdigitale clinical isolate D245	1.95	>250 (250^*a*^)	>250 (125^*a*^)	62.5
*Scopulariopsis brevicaulis* clinical isolate S65	1.95	31.25	15.62	3.91

a Weakening of fungal growth in comparison to growth control; no complete inhibition of growth.

### (ii) Toxicity to cell lines.

Mouse embryonic fibroblast (3T3-L1) lines and human dermal fibroblast (WS1) and skin keratinocyte (HaCaT) lines were used for toxicity studies. For the WS1 cell line, regardless of the presence (10%) or absence of serum for polymer concentrations up to 100 μg/mL, no decrease in viability below 50% of the control was observed (full data set in Fig. S5 and S6).

**FIG 2:**
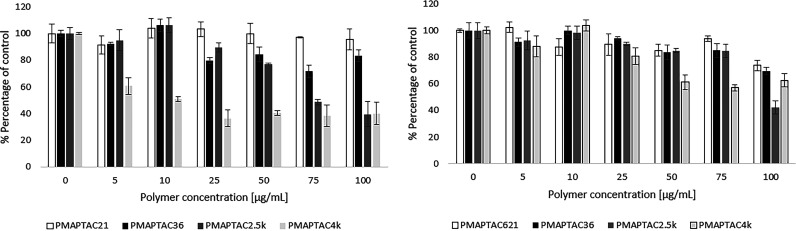
Effects of tested polycations on 3T3-L1 fibroblast survival in experiments without serum (left) and with serum (right) in the medium.

There was no statistically significant difference in effect between polymers of different molecular weights. The only noticeable difference between the test sets is the approximately 20% greater negative effect in the serum-free systems, which was to be expected due to the interaction of polycations with serum proteins. The high resistance of this cell line, which is not dependent on molecular weight, led to the use of the 3T3-L1 cell line to assess the toxicity of polymers and the influence of their physicochemical properties on this parameter. The obtained data are presented in Fig. 2. For serum and serum-free conditions, the greater the mass of polymer, the greater the negative effect on cells. For serum systems for the all concentrations tested, the number of viable cells did not fall below 50%. For the serum-free system, toxicity was slightly greater, and over the concentration range described here, a decrease in cell numbers of more than 50% occurred only for the two polymers with the highest masses (PMAPTAC4k and PMAPTAC2.5k for concentrations above 25 μg/mL and 75 μg/mL, respectively). For the polycation PMAPTAC4k, the number of cells in the concentration range of 25 to 100 μg/mL did not fall below 40%, which indicated that this polymer showed moderate toxicity toward these cells.

**FIG 3:**
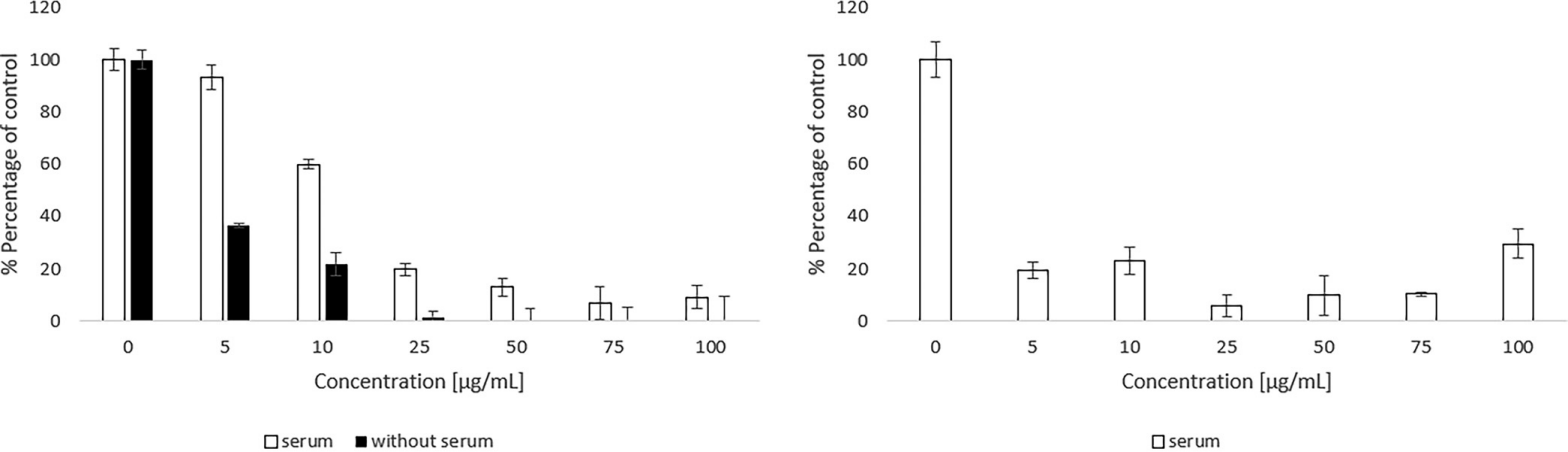
Effects of commercially used antifungal drugs on 3T3-L1 fibroblast survival: terbinafine (left) and ciclopirox (right).

Toxicity evaluation of two commercially used antifungal drugs, ciclopirox and terbinafine, was performed under analogous conditions (same cell line, medium composition, and exposure time). The obtained data for 3t3-L1 cells are presented in Fig. 3.

The graphs below (Fig. 2) show that all tested macromolecules were significantly less toxic than the low-molecular-weight drugs. For terbinafine, the concentration of 25 μg/mL caused a decrease in the number of cells to below 50% of control in serum medium, while an analogous effect in serum-free medium was caused by a dose of 5 μg/mL of the drug. The results for the second compound show that even concentrations of less than 5 μg/mL caused the death of more than half of the cells. In the case of ciclopirox, due to the strong serum interaction of this compound, an experiment in a serum-free medium would be unreliable and was not performed (https://go.drugbank.com/drugs/DB01188).

Considering the results obtained in antifungal activity testing and cytotoxicity studies and also practical considerations (the inconvenience of multistage synthesis additionally associated with more expensive reagents and the fact of obtaining greater mass dispersion for PMAPTAC2.5k), the polymer PMAPTAC4k was selected as the optimal leading compound for subsequent measurements. The effect of this polymer on keratinocytes (HaCaT) was additionally studied with up to 100 μg/mL in a medium containing 10% serum and in serum-free medium. For these concentrations, no effect on cell survival was recorded (full data set in Fig. S7). The effects of terbinafine and ciclopirox on this cell line were also investigated to obtain the full picture when it comes to comparing the toxicities of the new polymer and previous commercially used antifungal compounds. As expected, low-molecular weight compounds were less toxic to keratinocytes. Ciclopirox under serum conditions at a concentration of 5 μg/mL caused a decrease in the number of cells to about 70%, while from 10 to 100 μg/mL the decrease was to approximately 60%, regardless of the concentration (full data set in Fig. S8). Terbinafine, on the other hand, caused cell counts to fall below 50% only at concentrations above 25 μg/mL under serum and serum-free conditions (full data set in Fig. S9). Comparing the results obtained for antifungal drugs and PMAPTAC4k, it can be concluded that the polymer was also less toxic than commercially used antifungals in case of skin-modeling cells. This is key information to suggest a potential external application on the skin. Also important in this context is transdermal transport, which was excluded for this polymer using a diffusion chamber, as described in the next section.

### Franz diffusion chamber experiment.

The detection of the polymer in this experiment was possible due to the fluorescein labeling of the PMAPTAC2.5k polymer, which has a molecular weight similar to that of the PMAPTAC4k polymer (PMAPTAC4k labeling turned out to be unachievable), and fluorescence measurements. The time points used correspond to those for skin short-term exposure to the ointment/cream formulation (30 min and 90 min) and long-term exposure not interrupted by any hygienic activities (24 h). For all exposure times, comparing the membrane (Strat-M)-simulating skin barrier to a control that provides no barrier to diffusion (Isophore polycarbonate [PC] membrane with a 0.4-μm pore size), it can be concluded that the polymer will not penetrate the skin. The values of transfer of polymer mass via the skin-simulating barrier did not exceed the detection threshold (0.1 mg/L, which corresponds to 0.2% of total hypothetical polymer transfer mass), while in the system without the barrier transfer the values were 0.4, 15.4, and 98.0% after 30 min, 90 min, and 24 h. This difference is significant, and the result confirms the fact that for a macromolecule with high hydrophilicity, which is characteristic for polycation structure, any transdermal transport should not be expected.

### *Ex vivo* interaction with blood components.

**FIG 4:**
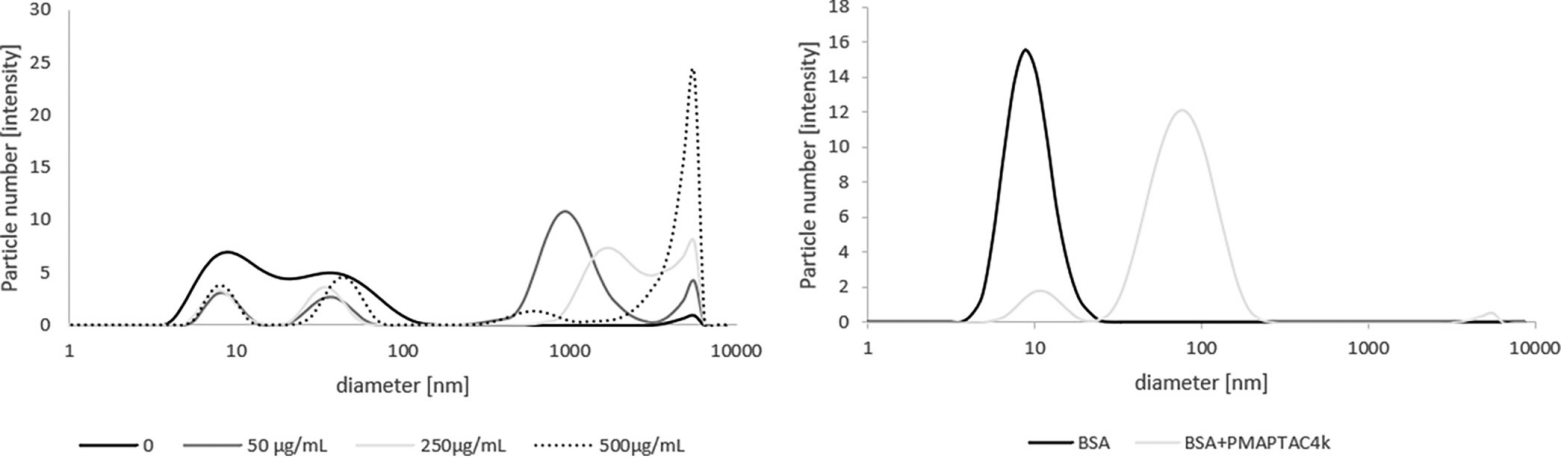
Diameters of the objects formed based on dynamic light scattering. (Left) Interaction of polymer with bovine serum and effect of concentration on the particles formed; (right) interaction of polymer with bovine serum albumin alone.

**FIG 5:**
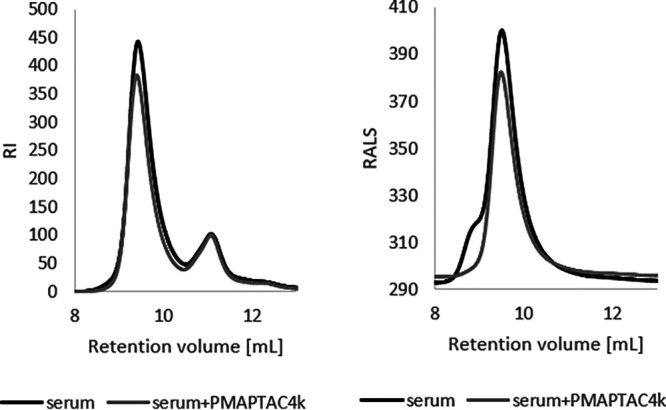
Effect of the polymer on serum composition after removal of the products of these interactions by gel permeation chromatography. RI, refractive index detector signal; RALS, right-angle light scattering detector signal.

**(i) Interaction with serum proteins.** The polymer interacts with serum proteins, and in our case, submicrometric particles were formed, but their size did not exceed 5 μm for concentrations below 250 μg/mL (Fig. 4, right). This was also supported by the antifungal activity of this compound in samples containing serum (higher polymer MIC values in the presence of serum). The processes that accompany the binding of serum proteins by polymers can affect the composition of this protein mixture. Therefore, an attempt was made to assess with which serum components the studied PMAPTAC4k polymer reacts. Performed studies were based on changes in serum composition after separation of the products of this reaction. The results obtained indicate that the polymer interacted with serum proteins and caused a decrease in the concentration of the most abundant proteins in the mixture and with the highest molecular weight, which was presumably albumin (as also shown by the dynamic light scattering [DLS] results [Fig. 4, left]). A decrease in signals for both detectors with the lowest retention times corresponding to the highest molecular weights was observed (Fig. 5). These were decreases of no more than 10% (based on the respective areas under the peak) and supported previous results (Fig. 4) confirming that the polymer interacts with serum proteins.

### (ii) Interaction with blood cells.

**FIG 6:**
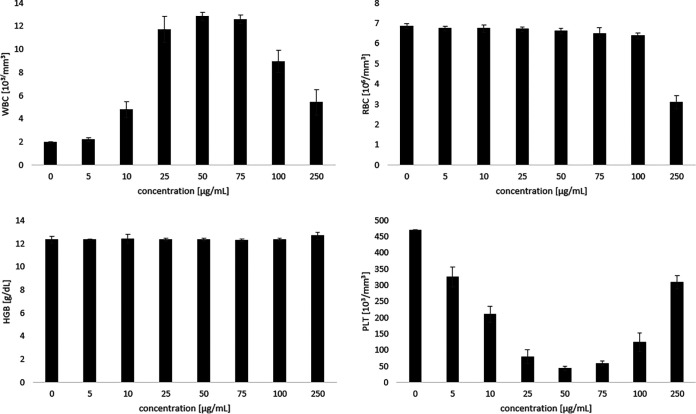
*In vitro* effect of the PMAPTAC4k polymer on complete blood counts. WBC, white blood cells; RBC, red blood cells; HGB, hemoglobin; PLT, blood platelets.

**FIG 7:**
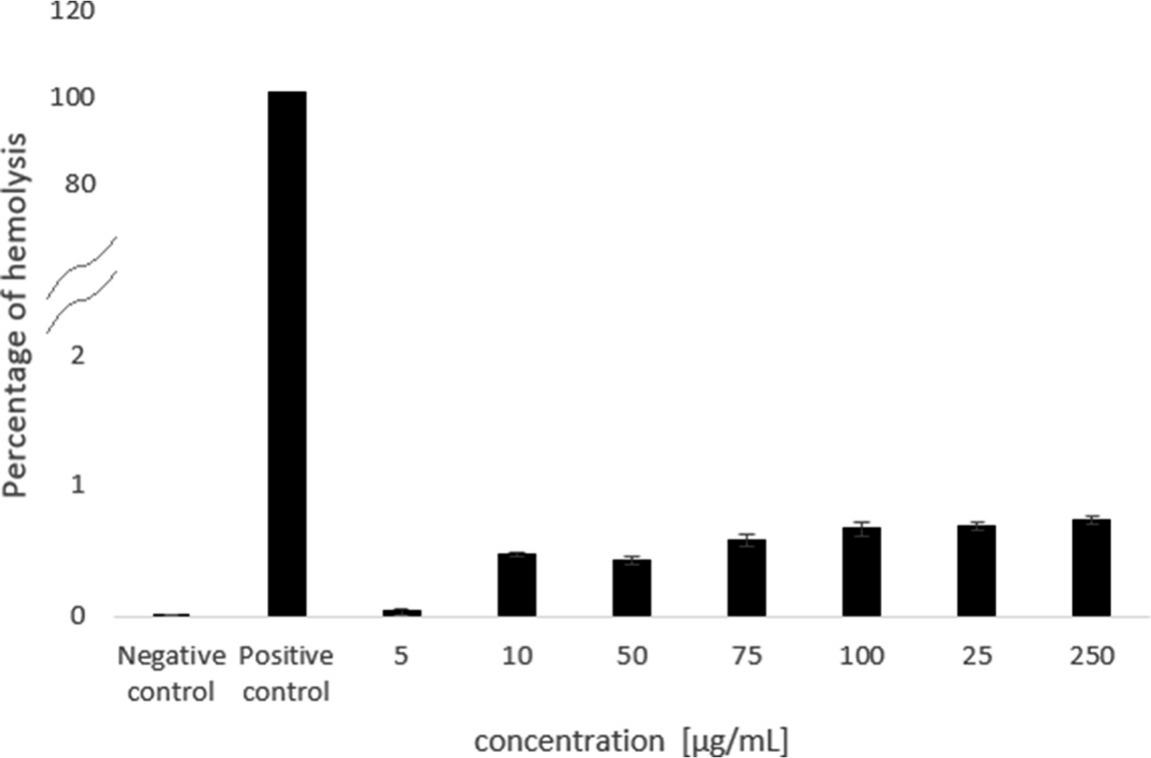
*In vitro* effect of the PMAPTAC4k polymer on hemolysis. Saline solution (0.9% NaCl) was employed as a negative control and distilled water as a positive control.

The number of blood cells, including red blood cells, white blood cells, and platelets, was measured after the incubation of whole blood with PMAPTAC4k using impedance technology. PMAPTAC4k significantly interacted with blood cells, as reflected mainly by changes in the number of white blood cells and platelets (Fig. 6). PMAPTAC4k exhibited no significant differences in red blood cell numbers at concentrations up to 100 μg/mL (Fig. 6). There was a decreased red blood cell number in the case of the highest concentration tested, 250 μg/mL, and an accelerated blood cell sedimentation (Fig. S10) after addition of the polymer, which may have been due to the significant aggregation of erythrocytes (19) which, however, was not accompanied by hemolysis (below 2%) within the concentration limits (Fig. 7).

## DISCUSSION

Fungal infections are diseases to which medicine, the pharmaceutical industry, and the media do not pay much attention and treat marginally. However, certain mycoses affect a large percentage of the general population. These include mycoses of the skin and its appendages, among which onychomycosis itself affects about 2 to 13% of the world’s population, especially middle-aged and elderly people (20% of patients aged 40 to 60 years and up to 50% of patients at the age of 70 years) (20).

The lesions in superficial mycoses often look unsightly, which causes embarrassment in an infected person. Some of those infections are contagious and require appropriate treatment to prevent spread of fungi to other people. Therefore, the consequence of superficial mycoses is a disturbance of not only physical health but also the mental state, interpersonal relations, and the quality of life. Currently, only a few antifungal substances are used to treat mycoses. There are also more and more difficulties in the treatment of these infections related to the more frequently reported drug resistance of fungi to antimycotics and the limitation of their use due to contraindications and side effects. High-level resistance to most currently available antimycotics has been demonstrated, among other things, for *Scopulariopsis* (21) and Fusarium (22, 23), which were included in the present research. Due to the growing problem of fungal infections, the search for new substances with antimycotic activity is a very important issue.

In this paper, the results of preliminary *in vitro* studies on the antifungal activity and toxicity of new polymers based on MAPTAC are presented. Special emphasis has been placed on the study of the action of polymers on fungi causing skin and nail mycoses and the toxicity of polycations for skin fibroblasts and keratinocytes. It was shown that antifungal activity and toxicity of the tested polymers are dependent on the molecular weight. Only polymers with a molecular weight above 0.5 mDa exhibited antifungal activity, but this was simultaneously associated with their greater toxicity.

One polymer with desirable antifungal properties and acceptable toxicity was selected. This polymer, PMAPTAC4k, is active against some yeasts and filamentous fungi. The antifungal spectrum includes species that are among the most common etiological factors of mycoses of keratinized tissues, i.e., Trichophyton interdigitale, Trichophyton mentagrophytes, Trichophyton rubrum, Trichophyton tonsurans, *Scopulariopsis brevicaulis*, and Fusarium species. The MIC values obtained were 0.98 to 1.95 μg/mL for *Trichophyton* species,1.95 to 3.91 μg/mL for *Scopulariopsis brevicaulis*, and 1.95 to 7.81 μg/mL for Fusarium species. *Trichophyton* species are the dominant etiological agents of skin and nail mycoses. *Scopulariopsis brevicaulis*, which exhibits natural resistance to many antifungal drugs (20), is among the most common nondermatophyte filamentous fungi causing onychomycoses. Moreover, these fungi are often the cause of mixed fungal infections in which the main pathogen is a dermatophyte and the mold infection appears secondarily, and such infections may not respond to standard treatment, as available antifungal drugs may be inactive (24, 25). Fusarium species are mainly associated with fungal keratitis but also have been reported as skin and nail pathogens. Fusarium infections are emerging because of multidrug resistance (22, 23). Information on acquired terbinafine resistance in *Trichophyton* has also been reported (26, 27). For this reason, information about the antifungal activity of the new polymer against the above-mentioned fungi and at the same time low toxicity to keratinocytes and fibroblasts is valuable. This compound may potentially be considered a new candidate for topically administered preparations in the case of skin and nail mycoses. It was also shown that the polymer has greater antimycotic activity than two commercially available drugs, terbinafine and ciclopirox, commonly used in the treatment of mycoses of the skin and its appendages. PMAPTAC4k MICs against S*copulariopsis brevicaulis*, Fusarium solani, Fusarium oxysporum, Trichophyton mentagrophytes, Trichophyton interdigitale, Trichophyton tonsurans, and Trichophyton rubrum were lower than those of ciclopirox. These concentrations were 3 times lower (for Trichophyton rubrum) to even 12 times lower (for Fusarium species). The new polymer also exhibited better activity than terbinafine against *Scopulariopsis brevicaulis* and Fusarium species: the obtained MIC values of the polymer were 12 to over 50 times lower. The activity against *Trichophyton* was comparable: the MICs of terbinafine ranged 0.19 to 0.78 μg/mL, while those for tested polymer were 1.95 to 3.91 μg/mL.

Experiments performed in this work to preliminarily assess the toxicity of the tested compounds showed that the negative effect on mammalian cells, similar to antifungal activity, increased with increasing molecular weight. However, the antifungal effect was stronger and for a mass of approximately 0.5 mDa, i.e., that of the PMAPTAC4k polymer, an optimal antimycotic effect and acceptable toxicity were achieved. A further increase in mass did not result in better antifungal properties but did result in increased toxicity to mammalian cells. This resulted in the choice of this polymer for further studies. The aim of those studies was to determine whether it would be possible to use this polymer intrinsically or whether only extrinsic use would be recommended. To achieve this, preliminary hemocompatibility studies, as an essential part of preclinical blood-contacting drug discovery (28), were carried out to assess the effects of PMAPTAC4k on blood morphology and the interaction with serum proteins. This direction of research was based on the fact that polymeric drugs are not absorbed from the gastrointestinal tract, so the potential route of administration would be parenteral. In the range of concentrations investigated, i.e., up to 250 μg/mL, the effect on blood morphotic elements was limited mainly to a negative effect on platelets and white blood cells. PMAPTAC4k decreased the number of platelets in a concentration-dependent manner up to 50 μg/mL. A reduction in the platelet count may be due to their adhesion and aggregation with positively charged polymer, as well as to the formation of platelet aggregates in the blood that can be counted as leukocytes. Indeed, a recently published report has demonstrated that positively charged dendrimers activate platelets, leading to their aggregation, and dramatically alter platelet morphology (29). This indicates the thrombogenic properties of the studied molecule. From a concentration of 75 μg/mL upwards, PMAPTAC4k increased the number of platelets. The most probable explanation for these results is that fragmented white and red blood cells were counted as platelets by an automated blood counter. For the highest concentration, a reduction in the number of red blood cells was observed, which had already been observed for compounds of this type (19).

The greatest danger of interaction of polymers with serum proteins (as often occurs with polycations [29]) is the formation of particles of micro- and submicrometric size as a result of these reactions, which could block the capillary blood vessels. In the case of PMAPTAC4k, the size of these products was evaluated using DLS. The conclusion from the results obtained is that the polymer interacts with serum proteins and in this case submicrometric particles are formed, but their size does not exceed 5 μm for concentrations below 250 μg/mL. That suggests that the risk of capillary congestion after intravenous administration at these concentrations should be considered low. These effects are indicative of moderate toxicity and suggest the extrinsic use of this polymer, which, after all, is the main mode of application of today’s known antifungal preparations. The overall results obtained in this study suggest that PMAPTAC4k can be considered a promising candidate for more advanced studies of new antifungal drugs.

There are no data on antifungal activity of PMAPTAC4k in the literature. These are the first studies of this type. It was shown that the range of action of the new polymer PMAPTAC4k is selective against some filamentous fungi but includes species that are among the most common causes of skin and nail mycoses and species that are considered to be multidrug resistant (*Scopulariopsis brevicaulis* and Fusarium species), and the MIC values against these strains are relatively low (0.98 to 7.81 μg/mL). The results obtained indicate that PMAPTAC4k may be an alternative to known drugs, e.g., ciclopirox and terbinafine, or a supplement to standard treatment. Understanding the mechanism of action of the polymer requires further research. The results obtained so far indicate a surface effect of the molecule, most likely on the fungal cell wall. This could explain the selective scope of the polymer’s action, as fungi are a group of organisms characterized by great morphological diversity, including the cell wall components (30).

## MATERIALS AND METHODS

### Materials.

The following materials were used: RPMI 1640 medium with l-glutamine and without sodium bicarbonate, Dulbecco’s modified Eagle’s medium (DMEM), fetal bovine serum, dimethyl sulfoxide (DMSO) (sterile filtered bioreagent, suitable for hybridoma, ≥99.7%), penicillin-streptomycin solution, 4,4′-azobis(4-cyanovaleric acid) (V-501), 4-cyano-4-(phenylcarbonothioylthio)pentanoic acid, MAPTAC monomer solution (50% in water), inhibitor removers (prepacked column for removing hydroquinone and monomethyl ether hydroquinone), Tween 20 (molecular biology grade; Sigma-Aldrich, St. Louis, MO, USA), glucose, methanol (analytical purpose purity), NaCl (analytical purpose purity), Na_2_SO_4_ (analytical purpose purity), acetic acid (99%; Chempur, Piekary Slaskie, Poland), 3-(*N*-morpholino)propanesulfonic acid (MOPS; Glentham Life, Corsham, UK), flat-bottom polypropylene 96-well microdilution plates (VWR, Radnor, PA, USA), Sabouraud glucose agar with chloramphenicol, neopeptone (Difco Laboratories Inc., Franklin Lakes, NJ, USA), glucose (Chempur), agar (Biocorp, Waszawa, Poland), chloramphenicol (Farm-Impex, Gliwice, Poland), Czapek yeast extract agar, ZnSO_4_·7H_2_O (POCH, Gliwice, Poland), CuSO_4_·7H_2_O (ACROS, Belgium), MgSO_4_·7H_2_O (POCH), KCl (Chempur), NaNO_3_ (POCH, Poland), saccharose (Chempur, Piekary Slaskie, Poland), K_2_HPO_4_ (Chempur), agar (Biocorp, Warsaw, Poland), yeast extract (Oxoid, Pratteln, Switzerland), ciclopirox olamine (99.34%; Pol-Aura, Poland), and terbinafine hydrochloride (99.9%; Thermo Scientific, Waltham, MA, USA).

### Apparatuses.

Apparatuses used included the following: densitometer (Biosan, Poland), incubator (POL-EKO, Poland), vortex mixer (Labnet, Poland), FTIR spectrometer (Nicolet iS10, SMART iTX; Thermo Scientific, Waltham, MA, USA), simultaneous CHNS combustion analyzer apparatus Micro Cube elemental analyzer (Elementar Vario, Langenselbold, Germany), Zeta sizer Nano ZS (Malvern Panalytical, Malvern, UK), microplate reader (Synergy HTX; BioTek, Winooski, VT, USA), and animal blood counter (ABC Vet; Horiba ABX, France).

### Cell cultures.

Cell lines 3T3-L1 (CL-173) and WS1 (CRL-1502) were both from the American Type Culture Collection (ATCC). The HaCaT cell line was obtained from Thermo Fisher Scientific.

### Fungal strains.

Fungal strains used in this study included strains from the American Type Culture Collection (ATCC), strains from German Collection of Microorganisms and Cell Cultures (DSMZ), and clinical isolates deposited at the Chair of Microbiology, Jagiellonian University Medical College, Cracow, Poland. The names of tested strains are included in the tables presenting the results of the research on the antifungal properties of polymers.

### Synthesis of polymers.

Syntheses of polycations of lower molecular weight were performed based on earlier work (31). First, the polymerization inhibitor was removed from the MAPTAC monomer solution (50% in water) by passing this liquid through neutralized alumina (inhibitor remover prepacked column). Then 6 mL of MAPTAC monomer solution, 15 mg of polymerization initiator V-501, and 5 mL of water were mixed. The mixture was heated to 40°C and degassed by passage through argon for 0.5 h. Then 34.66 mg of CPD ([4-cyano-4-(phenylcarbonothioylthio)pentanoic acid] was dissolved in 1 mL of methanol and added to the monomer solution. The reaction vessel was placed on a magnetic stirrer and stirred. The polymerization was carried out for 7 h (PMAPTAC62) or 24 h (PMAPTAC90) at 70°C. The solution containing the reaction product was then cooled. The next step was dialysis into distilled water in a dialysis tube (limiting mass throughput less than 3.5 kDa), which lasted for 5 days, with a change of water every 24 h. The polymer after dialysis was isolated from the solution by lyophilization.

Synthesis of PMAPTAC-macro-CTA (see below) was carried out in a manner similar to that described in the previous section. MAPTAC monomer solution (50% [wt/vol] in water) was passed through column inhibitor remover before use. Then 20 mL of MAPTAC (10 g), 19 mg of V-501 initiator, 100 mg of chain transfer agent [CTA; 4-cyano-4-(phenylcarbonothioylthio)pentanoic acid], and 3 mL of freshly distilled methanol (MeOH) were placed in a Schlenk flask and purged with argon for 0.5 h. The polymerization was carried out for 24 h at 70°C. The polymerization mixture was poured into a large excess of cold acetone to precipitate resulting polymer. The polymer was purified by reprecipitating from methanol into a large excess of acetone and dried in a vacuum oven at 50°C overnight. The obtained polymer (7.25 g; *M*_n_ = 11,200 Da; Ð = 1.06) could be used as a macro-CTA to prepare block copolymers.

For synthesis of PMAPTAC2.5k, PMAPTAC-macro-CTA (25 mg), MAPTAC monomer (2 mL, 50% water solution, passed through column inhibitor remover before use), initiator V-501 (0.2 mg), and 1 mL of freshly distilled MeOH were placed in a Schlenk flask and then purged with argon for 0.5 h. The polymerization was carried out for 24 h at 70°C. The polymerization mixture was poured into a large excess of cold acetone to precipitate resulting polymer. The polymer was purified by reprecipitation from methanol into a large excess of acetone and dried in a vacuum oven at 50°C overnight. For synthesis of fluorescent label PMAPTAC2.5k-b-F, PMAPTAC2.5k (250 mg), which can act as macro-CTA, initiator V-501 (0.15 mg), and fluorescein *o*-acrylate (5 mg) was dissolved in mixture of 50 mL of water and 2 mL of MeOH. The polymerization mixture was purged with argon for 0.5 h, and the polymerization was carried out for 24 h at 70°C. The solution was then cooled, and the crude product was purified by dialysis into distilled water (molecular weight cutoff = 3.5 kDa) for 4 days (water was changed 3 times a day) and isolated from the solution by lyophilization.

The final polymerization carried out in this work to obtain the largest polymer was based on the same ingredients as the earlier ones, except for CTA, which ensured that the classic radical polymerization proceeded. In the same way, the polymerization inhibitor was removed and then 6 mL of MAPTAC monomer solution, 15 mg of V-501 polymerization initiator, and 5 mL of water were mixed. The next step was analogous to the one described in the previous paragraph, and the only difference was that in both cases the synthesis was carried out for 4 h. Purification and isolation of polymers were also carried out as in the previous paragraph. For all polymers, FTIR spectra were measured, and elemental composition was evaluated using combustion analysis. Additionally, the molecular weight and its dispersion were determined using size exclusion/gel permeation (SEC/GPC) chromatography (details below).

### Determination of molecular weight of polymers by SEC/GPC.

The eluent consisted of 0.3 M Na_2_SO_4_ aqueous solution containing 0.5 M acetic acid, the flow rate was 0.4 mL/min, and the injection volume was 100 μL for all samples (31). The polymers were dissolved in the eluent and the concentration was 5 g/L. Due to the positive charge of these polymers (confirmed by measurements of the zeta potential), poly(2-vinylpyridine) standards were used for molecular mass determination. In all cases, the column set of PolySep-SEC GFC-P 2000, 4000, and 6000 liquid chromatography (LC) columns, 300 by 7.8 mm (Phenomenex, Torrance, CA, USA), was used.

### Testing of toxicity of polymers to cell lines.

3T3-L1 mouse embryo fibroblasts, WS-1 human skin fibroblasts, and HaCaT keratinocytes were used to assess the toxicity of polycations and commercial antifungal drugs (ciclopirox and terbinafine). The medium for those cell lines was Dulbecco’s modified Eagle’s medium, supplemented with fetal bovine serum for a final concentration of 10% (vol/vol) and 1% (vol/vol) penicillin-streptomycin solution. Cultures were incubated at 37°C in an atmosphere containing 5% of carbon dioxide (CO_2_). Cultures were seeded at 6 × 10^4^ cells per well in 24-well plates and grown for 24 h. After that, the medium was changed depending on the experiment to full serum (i.e., containing 10% fetal bovine serum) or serum free, and cells were treated with polycation solution (in serum-free medium) for the next 24 h to assess cytotoxicity using the crystal violet assay (32).

### Antimycotic properties of polymers.

An *in vitro* study of the antifungal properties of polymers was performed, and for the most active molecule (PMAPTAC4k), its antimycotic properties were compared with those of two antifungal drugs used in the treatment of infections, terbinafine and ciclopirox. The antifungal activity studies of the polymers and antimycotic drugs were performed with the microdilution method in a liquid RPMI 1640 medium with l-glutamine, without sodium bicarbonate, supplemented with 2% glucose and buffered to pH 7 with 4-morpholinepropanesulfonic acid (MOPS; 0.165 mol/L) in flat-bottom polypropylene 96-well microdilution plates, based on European Committee on Antimicrobial Susceptibility Testing methodology for fungi (https://www.eucast.org/astoffungi/methodsinantifungalsusceptibilitytesting/ast_of_moulds/, https://www.eucast.org/astoffungi/methodsinantifungalsusceptibilitytesting/susceptibility_testing_of_yeasts/) and the Hancock Lab procedure for cationic antimicrobial peptides (http://cmdr.ubc.ca/bobh/method/modified-mic-method-for-cationic-antimicrobial-peptides/).

Stock solutions of polycations were prepared in sterile distilled water to obtain concentrations of 5 g/L from which a series of 2-fold water dilutions were made. Antifungal drugs were dissolved in DMSO to obtain concentrations of 10 g/L. Then a 2-fold dilution series was prepared in DMSO, and finally, each concentration was diluted 1:10 in sterile distilled water. Wells 1 to 10 of each column of microdilution plates were filled with 20 μL of the corresponding concentration of tested polymers or antifungal drugs, while each well of columns 11 and 12 was filled with 20 μL of sterile distilled water or DMSO diluted 1:10 in sterile distilled water, for polymers and drugs, respectively.

Fungi were cultured on Sabouraud glucose agar with chloramphenicol, Czapek yeast extract agar, and potato dextrose agar to obtain optimal growth and sporulation. Yeast inocula were prepared by suspending a few representative colonies in sterile distilled water. Filamentous fungus colonies were covered with approximately 5 mL of sterile water supplemented with Tween 20, then the conidia were rubbed with a sterile cotton swab, and the suspension was transferred to a sterile tube attached to a sterile filter with a pore diameter of 10 μm. The suspension was filtered to remove hyphae and collected. For some strains, double filtration using filters with pore diameters of 20 μm and 10 μm was applied. Fungal suspensions were homogenized with a gyratory vortex mixer, and the cell density was adjusted to 0.5 McFarland. Then the inocula were diluted 1:20 in RPMI 1640 medium with l-glutamine, without sodium bicarbonate and with 2% glucose, buffered to pH 7 with MOPS (0.165 mol/L). This gave the following inoculum densities: 0.5 × 10^5^ to 2.5 × 10^5^ CFU/mL for yeasts and 1 × 10^5^ to 2.5 × 10^5^ CFU/mL for filamentous fungi.

The microdilution plates were inoculated with 180 μL of the fungal suspensions, except sterility control wells, which contained 180 μL of microbe-free RPMI 1640 with l-glutamine, without sodium bicarbonate and with 2% glucose, buffered to pH 7 with MOPS (0.165 mol/L). The range of final concentrations of tested polymers and antifungal drugs on microplates after the addition of the fungal suspension were 0.49 to 250 μg/mL and 0.19 to 100 μg/mL, respectively. The plates were incubated without agitation at 37°C (yeasts) or 27°C (molds and dermatophytes) in ambient air for 24 to 72 h. The antifungal activity was estimated by determining the MIC values, which were defined as no visible growth of fungi by the eye (visual readings). For yeast, an automatic reading was additionally performed with a microdilution plate reader (Tecan; Sunrise) by measuring the absorbance at a wavelength of 530 nm. The lowest concentration of the tested compound causing at least 95% growth inhibition in comparison to the growth control was defined as the MIC (automatic readings).

### Influence of serum on the antifungal activity of polymers.

The study of the effect of serum on antifungal activity was performed for the PMAPTAC4k polymer, for which the best antifungal activity and relatively low toxicity to mammalian cells were obtained. The test was performed according to the above-described procedure. Liquid RPMI 1640 medium with l-glutamine, without sodium bicarbonate and with 2% glucose, buffered to pH 7 with MOPS (0.165 mol/L) supplemented with fetal bovine serum (the same as used for cell line research) was used for culture of the fungi in the presence of PMAPTAC4k polymer. The serum was added in the following proportions to the medium: 20% medium plus 80% serum, 50% medium plus 50% serum, and 80% medium plus 20% serum. The MIC values of the polymer against selected tested strains, defined as no visible growth of fungi by eye, were determined.

### *Ex vivo* study on the interaction of selected polycations with blood.

Interaction of polycations with blood serum proteins was studied using dynamic light scattering (DLS) and gel permeation chromatography. To evaluate the creation of protein-polymer adducts in serum-polycation systems, mixtures of equal volumes of serum (additionally purified from submacroscopic protein aggregates by centrifugation at 10,000 rpm for 5 min) and PMAPTAC4k solutions in 0.1 M NaCl were prepared. DLS was then measured for these samples to assess the size of the resulting objects as a function of polymer concentration. An analogous measurement was also carried out for equal volumes of 1 g/L of polymer and 1 g/L of bovine serum albumin. These experiments were designed to indicate whether this protein also participates in reactions with polycation that may occur in serum. The study was performed only for PMAPTAC4k because this polymer showed the most promising antimycotic properties. The measurement of particle diameter was performed using a Zeta sizer Nano ZS. The data obtained using DLS were complemented by GPC measurements.

The eluent was a solution of 0.1 M NaCl in water, the flow rate was 0.8 mL/min, and the injection volume was 100 μL. The sera used were centrifuged at 10,000 rpm for 5 min in the same way as for the DLS measurements. Then 0.05 mL of the resulting supernatant was mixed with 0.95 mL of eluent, and this sample was used as a reference. The proper sample was a mixture of 0.5 mL of centrifuged serum and 0.5 mL of a 5-g/L solution of the polymer in the eluent, which was centrifuged (5 min and 10,000 rpm) and then diluted 10 times with the eluent. This sample was centrifuged again (5 min and 10,000 rpm) before injection. In all cases, a PolySep-SEC GFC-P linear column and LC column (300 by 7.8 mm) (Phenomenex, Torrance, CA, USA) were used.

### Zeta potential measurements of obtained polycations.

The measurement of the zeta potential was performed using a Zetasizer Nano ZS (apparatus and polymer solutions in demineralized water [pH 6.0; conductivity, 0.5 μS]) at a concentration of 5 g/L.

### Franz diffusion chamber.

The experiments were conducted using a 20-mm clear jacketed vertical glass Franz cell with a flat ground joint and 5-mL receptor volume. The formulation for which measurements were performed in addition to the fluorescently labeled polymer PMAPTAC2.5k-b-F (0.25 mg) contained hydroxyethylcellulose (50 mg) and glycerine (100 mg) and water in an amount such that the total mass of gel formed from the components applied to the membrane was 1 g. Time stamps of 30 and 90 min and 24 h were used. Fluorescence spectra of the acceptor solution (phosphate-buffered saline [PBS]; total volume of 5 mL) were recorded after each time stamp. The experiments were carried out at 37°C using a Strat-M membrane as a predictor of diffusion in human skin, and an Isophore PC membrane 0.4 μm in pore size as a positive control of no barrier to diffusion.

### Interaction with blood cells.

Blood samples were freshly collected from 8-week-old male Wistar rats (Centre of Experimental Medicine, Medical University of Bialystok, Poland) into test tubes containing 3.13% sodium citrate. For hemolysis studies, red blood cells were immediately separated by centrifugation (1,500 rpm, 10 min). Cells were then washed 3 times and diluted with saline solution (0.9% NaCl) to obtain a stock dispersion with a fixed concentration of hemoglobin (50% hematocrit). Fifty microliters of the red blood cell stock dispersion was added to 950 μL of saline solution containing different concentrations of PMAPTAC4k (5, 10, 25, 50, 75, 100, and 250 μg/mL) and incubated in an Eppendorf Thermomixer (Germany) at a mixing frequency of 450 rpm and a temperature of 37°C for 1 h. Intact red blood cells were removed by centrifugation at 10,000 rpm for 5 min. Then the absorbance of the resulting supernatant was measured at 540 nm using a microplate reader. Saline solution was employed as a negative control (0% lysis) and distilled water as a positive control (100% lysis). The hemolysis rate was calculated according to the following equation:

$$\text{Hemolysis rate }\left(\text{\%}\right)=\frac{D_{t}-D_{nc}}{D_{pc}-D_{nc}}\times 100$$

where *D_t_*, *D_nc_*, and *D_pc_* are the absorbances of the tested sample, negative control, and positive control, respectively.

For hematological studies, 900 μL of the citrate-anticoagulated blood was added to 100 μL of saline solution containing different concentrations of PMAPTAC4k (5, 10, 25, 50, 75, 100, and 250 μg/mL), mixed manually, and incubated for 10 min. Hematological parameters were measured using an animal blood counter.

### Statistics.

For the toxicity testing of polymers on cell lines, zeta potential measurements, and hemolysis and hematological studies, three independent measurements were made for each concentration/sample, and the result presented is the arithmetic mean ± standard deviation (SD). The studies of antifungal activity were performed in three replicates. Tables 2 to 5 show the range of MIC values obtained.
