# Supplementary material for: Studies on Antifungal Properties of Methacrylamido Propyl Trimethyl Ammonium Chloride Polycations and Their Toxicity In Vitro
Source: Microbiol Spectr. 2023 May 11;11(3):e00844-23. doi: 10.1128/spectrum.00844-23 (PMC10269872; doi:10.1128/spectrum.00844-23)
Supplement: Supplemental file 1 — Tables S1 to S3 and Fig. S1 to S10. Download spectrum.00844-23-s0001.docx, DOCX file, 0.4 MB [file spectrum.00844-23-s0001.docx]

**Supplementary material**

**Studies on antifungal properties of methacrylamido propyl trimethyl ammonium chloride polycations and their toxicity *in vitro***

Magdalena Skóra^1^, Magdalena Obłoza^3^, Małgorzata Tymecka^4^, Bartłomiej Kalaska^2^, Magdalena Gurgul^3^, Kamil Kamiński^3*^

^1^Department of Infections Control and Mycology, Chair of Microbiology, Jagiellonian University Medical College, Czysta 18 St., 31-121 Kraków, Poland

^2^Department of Pharmacodynamics, Medical University of Białystok, Mickiewicza 2c St., 15-089 Białystok, Poland

^3^Faculty of Chemistry, Jagiellonian University, Gronostajowa 2 St., 30-387 Kraków, Poland

^4^ Doctoral School of Exact and Natural Sciences, Faculty of Chemistry, Jagiellonian University, Gronostajowa 2 St., 30-387 Kraków, Poland

Table S1 Elemental composition of polymers obtained

| Name of polymer | N | C | H | S | N/C |
| --- | --- | --- | --- | --- | --- |
| PMAPTAC21 | 10.24 | 44.30 | 9.72 | 0 | 0.2311 |
| PMAPTAC36 | 10.58 | 45.61 | 9.99 | 0 | 0.2319 |
| PMAPTAC2.5k | 9.81 | 47.34 | 9.63 | 0 | 0.2072 |
| PMAPTAC4k | 10.45 | 47.07 | 9.94 | 0 | 0.2219 |

Table S2 The percentage of yeasts growth in the presence of PMAPTAC4k in relation to the control growth without the addition of the polymer determined in authomatic readings.

|  | PMAPTAC4k concentrations [µg/mL] | | | | | | | | | |
| --- | --- | --- | --- | --- | --- | --- | --- | --- | --- | --- |
|  | 250 | 125 | 62.5 | 31.25 | 15.62 | 7.81 | 3.91 | 1.95 | 0.98 | 0.49 |
| Candida albicans ATCC 90028 | ≥ 90% | ≥ 90% | ≥ 90% | ≥ 90% | ≥ 90% | ≥ 90% | ≥ 90% | ≥ 90% | ≥ 90% | ≥ 90% |
| Candida auris DSM 21092 | 75% | 65% | 60% | 67% | 76% | 65% | 76% | 86% | 83% | ≥ 90% |
| Candida glabrata ATCC 15454 | ≥ 90% | ≥ 90% | 89% | 89% | ≥ 90% | ≥ 90% | ≥ 90% | ≥ 90% | ≥ 90% | ≥ 90% |
| Candida krusei ATCC 6258 | 75% | 64% | 71% | 71% | 70% | 82% | 72% | 76% | ≥ 90% | ≥ 90% |
| Candida tropicalis ATCC 1369 | ≤ 5% | ≤ 5% | ≤ 5% | ≤ 5% | 10% | 36% | 83% | ≥ 90% | ≥ 90% | ≥ 90% |
| Candida parapsilosis DSM 5784 | ≤ 5% | ≤ 5% | ≤ 5% | ≤ 5% | ≤ 5% | ≤ 5% | ≤ 5% | 17% | 56% | 85% |
| Cryptococcus neoformans ATCC 204092 | ≤ 5% | ≤ 5% | ≤ 5% | ≤ 5% | ≤ 5% | ≤ 5% | ≤ 5% | ≤ 5% | 8% | 67% |
| Cryptococcus neoformans DSM 6973 | ≤ 5% | ≤ 5% | ≤ 5% | ≤ 5% | ≤ 5% | ≤ 5% | ≤ 5% | ≤ 5% | 13% | 47% |
| Cryptococcus neoformans clinical isolate EN | ≤ 5% | ≤ 5% | ≤ 5% | ≤ 5% | ≤ 5% | ≤ 5% | ≤ 5% | ≤ 5% | ≤ 5% | 36% |

Table S3 The percentage of yeasts growth in the presence of PMAPTAC2.5k in relation to the control growth without the addition of the polymer determined in authomatic readings.

|  | PMAPTAC2.5k concentrations [µg/mL] | | | | | | | | | |
| --- | --- | --- | --- | --- | --- | --- | --- | --- | --- | --- |
|  | 250 | 125 | 62.5 | 31.25 | 15.62 | 7.81 | 3.91 | 1.95 | 0.98 | 0.49 |
| Candida albicans ATCC 90028 | ≥ 90% | ≥ 90% | ≥ 90% | ≥ 90% | ≥ 90% | ≥ 90% | ≥ 90% | ≥ 90% | ≥ 90% | ≥ 90% |
| Candida auris DSM 21092 | 80% | 47% | 32% | 30% | 58% | 63% | 59% | 67% | 76% | ≥ 90% |
| Candida glabrata ATCC 15454 | ≥ 90% | ≥ 90% | ≥ 90% | ≥ 90% | ≥ 90% | ≥ 90% | ≥ 90% | ≥ 90% | ≥ 90% | ≥ 90% |
| Candida krusei ATCC 6258 | 70% | 58% | 49% | 53% | 61% | 69% | 68% | 72% | 69% | ≥ 90% |
| Candida tropicalis ATCC 1369 | ≤ 5% | ≤ 5% | ≤ 5% | ≤ 5% | 28% | ≥ 90% | ≥ 90% | ≥ 90% | ≥ 90% | ≥ 90% |
| Candida parapsilosis DSM 5784 | ≤ 5% | ≤ 5% | ≤ 5% | ≤ 5% | ≤ 5% | 26% | 39% | 48% | 58% | 82% |
| Cryptococcus neoformans ATCC 204092 | nd | nd | nd | nd | nd | nd | nd | nd | nd | nd |
| Cryptococcus neoformans DSM 6973 | ≤ 5% | ≤ 5% | ≤ 5% | ≤ 5% | ≤ 5% | 16% | 22% | 40% | 58% | 88% |
| Cryptococcus neoformans EN | ≤ 5% | ≤ 5% | ≤ 5% | ≤ 5% | ≤ 5% | ≤ 5% | ≤ 5% | ≤ 5% | ≤ 5% | 17% |


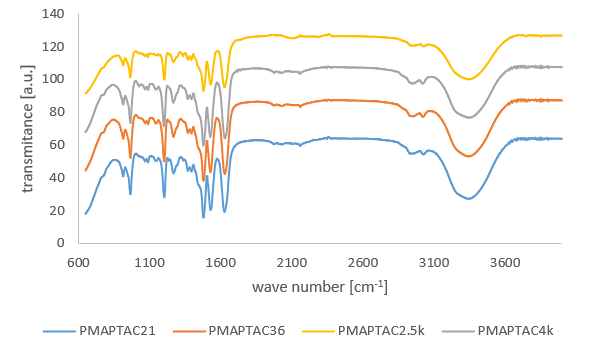


Figure S1 FT-IR spectra of the investigated polymers.


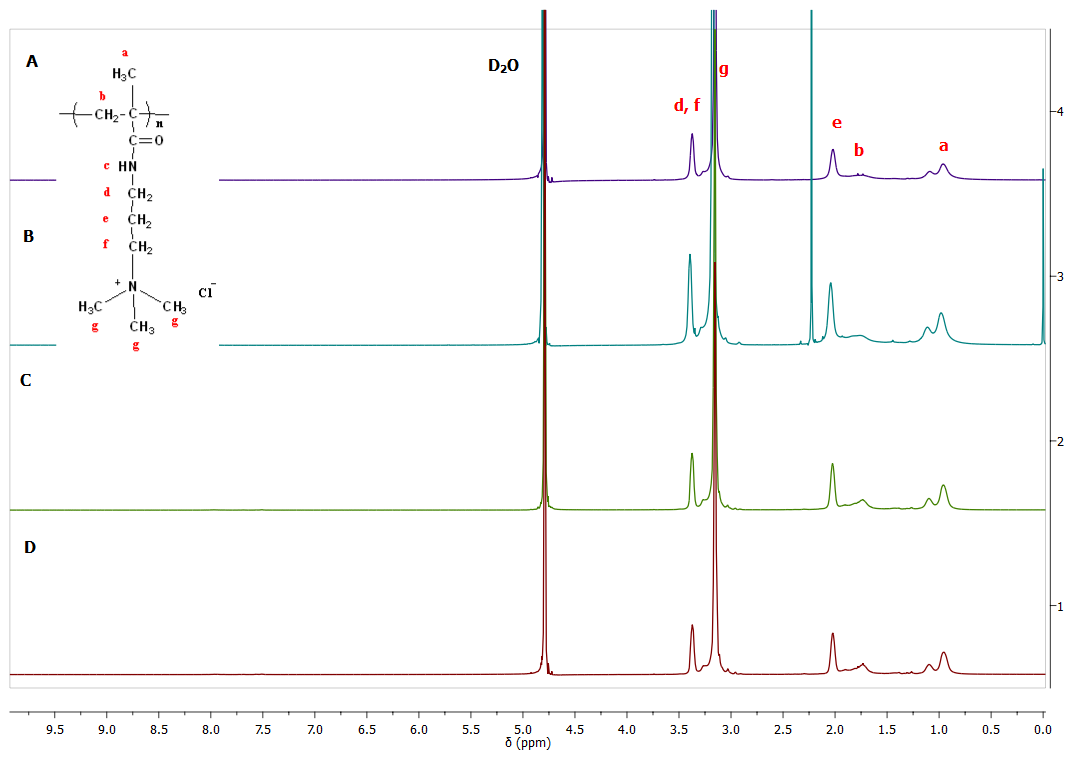


Figure S2 ^1^H NMR spectra of the investigated polymer in D2O: A-PMAPTAC4k, B-PMAPTAC2.5k, C-PMAPTAC36, D-PMAPTAC21.


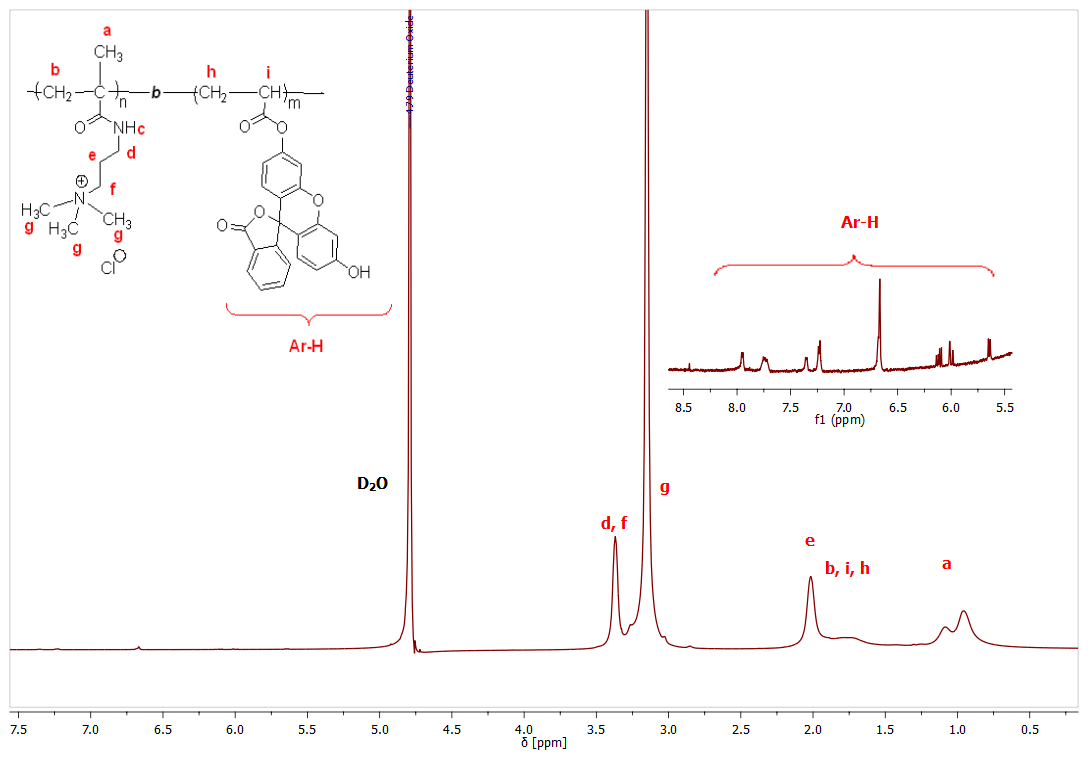


Figure S3 ^1^H NMR spectra of the fluorescent labeled polymer in D_2_O


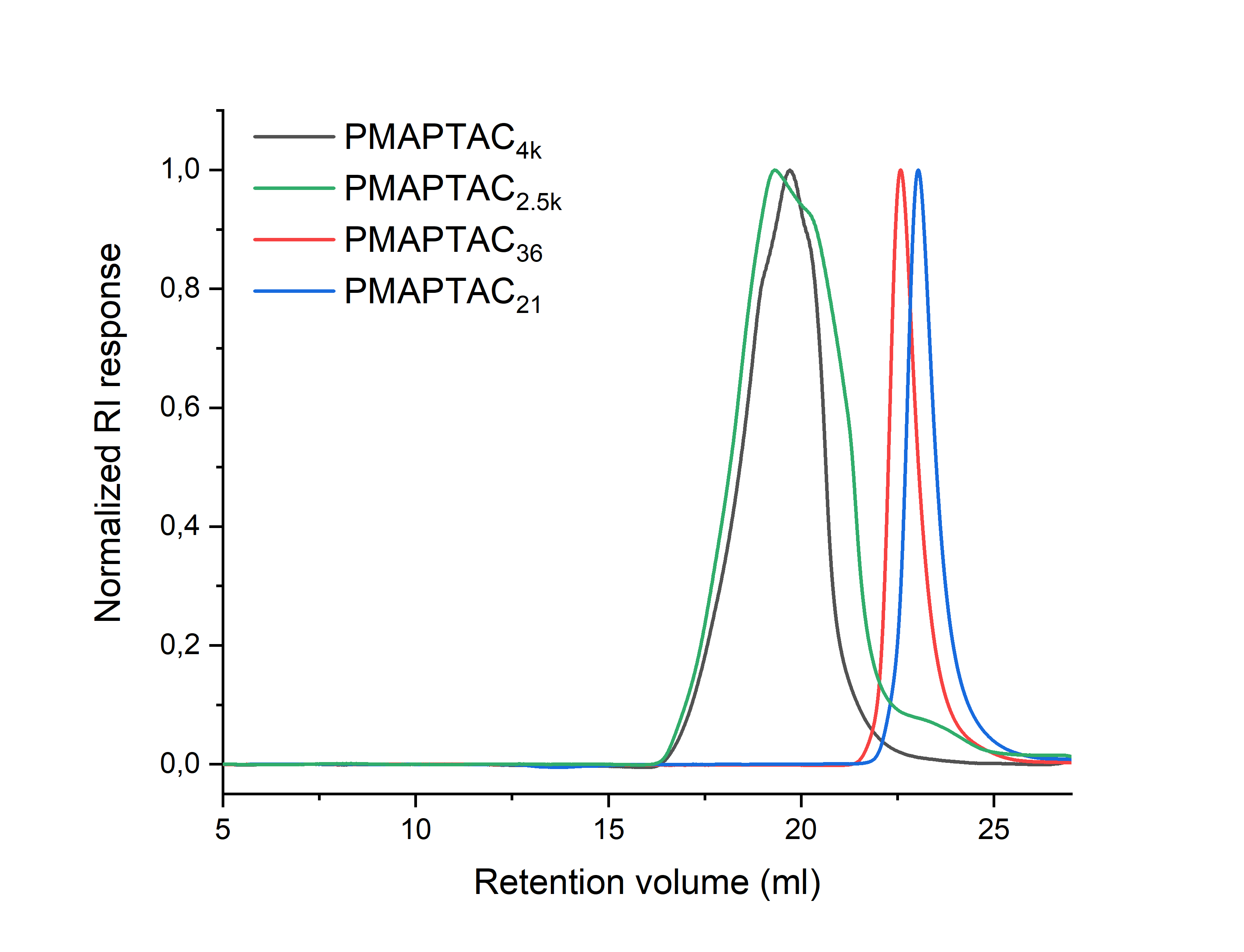


Figure S4. ^­^Normalized SEC/GPC chromatographs of investigated polymers


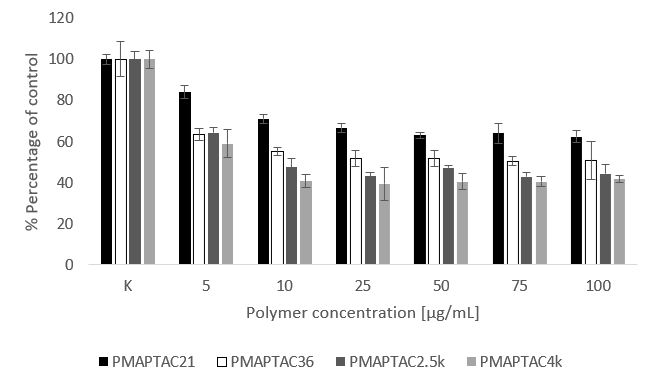


Figure S5. PMAPTAC polymers toxicity experiment in serum-free medium on normal human skin cells WS1 (ATCC CRL-1502).


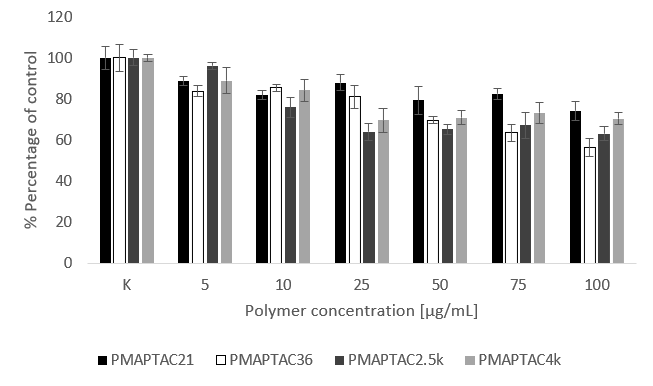


Figure S6. PMAPTAC polymers toxicity experiment with medium containing 10% fetal bovine serum on normal human skin cells WS1 (ATCC CRL-1502).


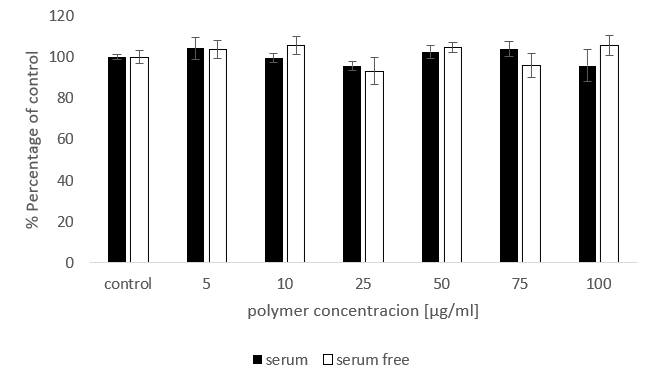


Figure S7. PMAPTAC4k toxicity on keranocytes (HaCaT). Experiment with medium containing 10% fetal bovine serum and serum free.


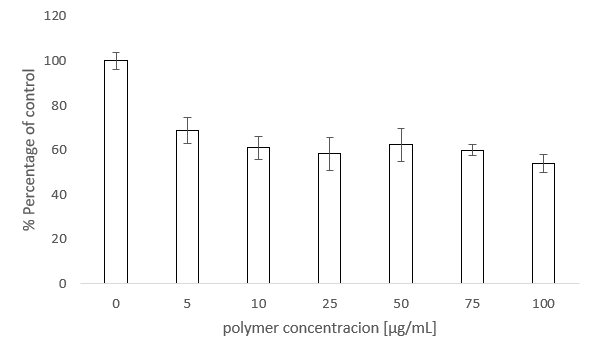


Figure S8. Ciclopirox toxicity on keranocytes (HaCaT). Experiment with medium containing 10% fetal bovine serum.


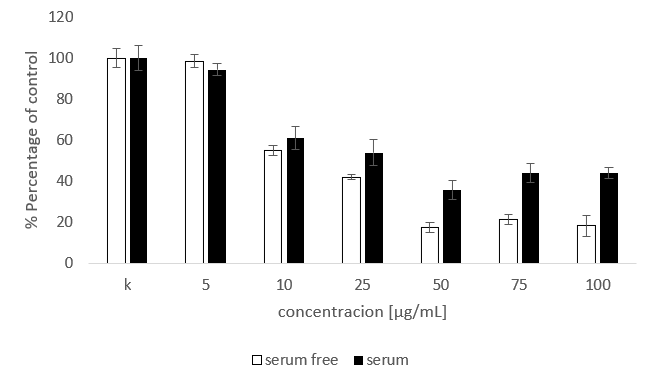


Figure S9. Terbinafine toxicity on keranocytes (HaCaT). Experiment with medium containing 10% fetal bovine serum and serum free.


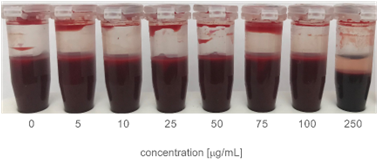


Figure S10. Effect of PMAPTAC4k on rat blood cell sedimentation. Nine hundred µL of citrate-anticoagulated rat blood was added to 100 µL of saline solution containing different concentrations of PMAPTAC4k and mixed manually. A representative picture was taken after 1 hour of incubation at room temperature.
